# The lncRNA lnc_AABR07044470.1 promotes the mitochondrial-damaged inflammatory response to neuronal injury via miR-214-3p/PERM1 axis in acute ischemic stroke

**DOI:** 10.1007/s11033-024-09301-2

**Published:** 2024-03-11

**Authors:** Meng Wang, Hong Li, Yulin Qian, Shanshan Zhao, Hao Wang, Yu Wang, Tao Yu

**Affiliations:** 1 First Teaching Hospital of Tianjin University of Traditional Chinese Medicine, Tianjin, 300380 People’s Republic of China; 2 National Clinical Research Center for Chinese Medicine Acupuncture and Moxibustion, Tianjin, 300380 People’s Republic of China

**Keywords:** AIS, Lnc_AABR07044470.1, MiR-214-3p, PERM1, Inflammatory response, Neuronal injury

## Abstract

**Purpose:**

We investigated the role of lnc_AABR07044470.1 on the occurrence and development of acute ischemic stroke (AIS) and neuronal injury by targeting the miR-214-3p/PERM1 axis to find a novel clinical drug target and prediction and treatment of AIS.

**Methods:**

The mouse AIS animal model was used in vivo experiments and hypoxia/reoxygenation cell model in vitro was established. Firstly, infarction volume and pathological changes of mouse hippocampal neurons were detected using HE staining. Secondly, rat primary neuron apoptosis was detected by flow cytometry assay. The numbers of neuron, microglia and astrocytes were detected using immunofluorescence (IF). Furthermore, binding detection was performed by bioinformatics database and double luciferase reporter assay. Lnc_AABR07044470.1 localization was performed using fluorescence in situ hybridization (FISH).Lnc_AABR07044470.1, miR-214-3pand PERM1mRNA expression was performed using RT-qPCR. NLRP3, ASC, Caspase-1 and PERM1 protein expression was performed using Western blotting. IL-1β was detected by ELISA assay.

**Results:**

Mouse four-vessel occlusion could easily establish the animal model, and AIS animal model had an obvious time-dependence. HE staining showed that, compared with the sham group, infarction volume and pathological changes of mouse hippocampal neurons were deteriorated in the model group. Furthermore, compared with the sham group, neurons were significantly reduced, while microglia and astrocytes were significantly activated. Moreover, the bioinformatics prediction and detection of double luciferase reporter confirmed the binding site of lnc_AABR07044470.1 to miR-214-3p and miR-214-3p to Perm1. lnc_AABR07044470.1 and PERM1 expression was significantly down-regulated and miR-214-3pexpression was significantly up-regulated in AIS animal model in vivo. At the same time, the expression of inflammasome NLRP3, ASC, Caspase-1 and pro-inflammatory factor IL-1β was significantly up-regulated in vivo and *in vitro.* The over-expression of lnc_AABR07044470.1 and miR-214-3p inhibitor could inhibit the neuron apoptosis and the expression of inflammasome NLRP3, ASC, Caspase-1 and pro-inflammatory factor IL-1β and up-regulate the expression of PERM1 in vitro. Finally, over-expression of lnc_AABR07044470.1 and miR-214-3p inhibitor transfected cell model was significant in relieving the AIS and neuronal injury.

**Conclusion:**

Lnc_AABR07044470.1 promotes inflammatory response to neuronal injury via miR-214-3p/PERM1 axis in AIS.

## Introduction

Acute ischemic stroke (AIS) is one of the most common cerebrovascular diseases, leading to severe disability and mortality. Neuronal death, neuro-inflammation and destruction of neurovascular units caused by AIS lead to severe neurological symptoms [1]. Timely restoration of cerebral blood flow perfusion and neuro-protective treatment are the main goals of AIS treatment. Unfortunately, effective treatments for AIS are limited and only intravenous thrombolytic therapy is recognized as an effective treatment. At present, although treatment measures have been developed for AIS, the benefits of timely reperfusion have been understood and thrombolytic therapy has become the basis of the current treatment of AIS,unfortunately, the effective treatment and clinical benefits of AIS are still not optimistic.

Immune and inflammatory responses are considered to be the key factors involved in the pathophysiology of AIS, which are closely related to the complex pathological changes of AIS [2, 3]. The inflammatory response occurs very rapidly within hours after stroke, and activation of neuro-immunity can directly affect the initiation, proliferation, and recovery stages of ischemic brain injury [4]. The specific mechanisms of inflammatory injury in stroke include: cerebral ischemia induces a large number of activation of innate glial cells (mainly microglia and astrocytes) in the central nervous system (CNS), and a variety of pro-inflammatory factors, such as IL-1β, IL-6 and TNF-α, released by glial cells directly aggravate the nerve injury [5, 6]. Many studies have suggested that the promotion of inflammation in the CNS could enlarge the area of cerebral infarction and aggravate the neurological injury in animal models of AIS [5, 7].Therefore, for the treatment of AIS, intervention of the inflammatory response in the CNS can be regarded as a feasible alternative treatment.

In recent years, long non-coding RNA (lncRNA) has gotwidely attention and research from many aspects in biomedical field. In the study of many diseases, some lncRNAs with functions of diagnosis and prognosis have gradually emerged [8, 9]. The lncRNA lnc_AABR07044470.1 is a hot star molecule in recent years. It is expressed in many normal tissues and the expression level is also strictly regulated, which is extremely important for embryo development, organism growth and development [10]. In many types of cerebrovascular diseases, lnc_AABR07044470.1 can play a role in occurrence and development of cerebrovascular diseases [11]. However, unfortunately, the role of lnc_AABR07044470.1 in AIS has not yet been revealed.

In recent years, more and more studies have shown that microRNA (miRNA) expression disorders exist in a variety of cerebrovascular diseases such as AIS, and it is closely related to the prognosis and treatment response of cerebrovascular diseases, suggesting that miRNA may be involved in every process of cerebrovascular disease occurrence and development [12, 13]. Multiple studies have reported that the expression of miR-214-3p is up-regulated in cerebral palsy, cerebral blood clot and Alzheimer’s disease [14]. However, at present, the relationship between miR-214-3p and AIS is not clear, and no studies have been reported at home and abroad.

The PGC1 and ESRR induced regulator, muscle 1 (PERM1) can positively regulate the expression of peroxisome proliferator-activated receptor γ coactivator 1α (PGC-1α) and ESRRα target genes, and play a role in glucolipid metabolism, energy transfer, mitochondrial biogenesis and oxidation response. Mitochondrial dysfunction can lead to serious impairment of cellular energy conversion, the most obvious manifestation is the activation of inflammatory response and the release of inflammatory factors. At the same time, linear correlation analysis showed that the decreased expression level of PERM1 was directly proportional to the number of neurons. It was speculated that the possible mechanism was that the decreased level of mitochondrial antioxidant components and uncoupling proteins caused by the decreased expression level of PERM1, which eventually led to the loss of neurons and neuro-degeneration [15]. In conclusion, increasing the expression level of PERM1in neurons can produce significant neuro-protective effects. However, the effect of increasing PERM1 in neurons on AIS and specific molecular mechanism are still unclear. Therefore, a better description of the molecular mechanism of PERM1 is of great significance for the treatment of AIS.

In this study, we predicted the relationship between lnc_AABR07044470.1 and miR-214-3p, or miR-214-3p and PERM1in AIS by bioinformatics database. Furthermore, the correlation between the function and regulatory of AIS and the expression level of lnc_AABR07044470.1, miR-214-3p and PERM1were further analyzed. Finally, experiments related to cell and animal models were established to study the role of lnc_AABR07044470.1, miR-214-3p and PERM1 in AIS, and to determine the target genes and related mechanism. In order to find more effective clinical prediction and treatment of AIS to provide the clinical basis and novel treatment targets.

## Materials and methods

### Animal model establishment and grouping

In this study, SPF C57BL/6J mice (20-25 g, purchased from Beijing Vital River Laboratory Animal Technology Co., Ltd., China) were fed in SPF environmental conditions of half day light and half day dark, temperature was 22 ± 2 °C and humidity was 60%±10%. The 50 C57BL/6J mice were randomly divided into sham and model groups. The sham group had 10 mice, and the remaining 40 mice in the model group. Global cerebral ischemia stroke model was prepared by four-vessel occlusion [16]: bilateral common carotid arteries were separated by a midneck incision after rats anesthesia, and the first transverse pterygous foramen was exposed in the posterior occipital position. The passing vertebral arteries under the first transverse pterygous foramen were thermo-coagulated for 2-4s for permanent occlusion. 24 h later, both common carotid arteries were clipped for 20 min under the awake state of the mice, and then permanent perfusion was performed. The criteria for the success of the animal model was as follows: the mice were in coma within 30-60s of ischemia, bilateral pupil dilation, and the righting reflex disappeared. In sham group, only blood vessel isolation and exposure were performed, without vertebral artery electrocoagulation and common carotid artery clipping.

Brain tissues were collected at 1, 3 and 7 days after modeling and a portion of the brain tissues was placed in 4% paraformaldehyde (PFA) and the rest was frozen in liquid nitrogen and stored at −80 °C in preparation for subsequent experiments. The experimental mice followed the regulations of the Experimental Animal Ethics Committee of the Ministry of Science and Technology and the Laboratory Management Regulations of the Animal Center of the Institute of Radiation Medicine, Chinese Academy of Medical Sciences (Approval NO.: IRM-DWLL-2,021,167).

### Isolation and culture of primary neurons

The mice were sterilized by 75% alcohol and then the brain tissues were slowly removed on a clean hood, and the cerebellum, brainstem, dura mater, pia mater and blood vessels were removed on ice. Transfering the remaining brain tissues to a petri dish that had been added with Hank’s buffer, and cut up the brain tissues. After Hank’s buffer was removed by pipetting, 3 mL trypsin was added and digested in cell incubator for 10 min. The samples were blew gently and added 1 mL DNase to tissue digestion. The tissue digestion was terminated by adding 3 mL DMEM complete medium. For the isolation and culture of primary neurons, the resuspended cells were added to poly-lysine coated petri dishes containing high glucose DMEM medium and incubated in the 37℃ incubator for 4 h. The high glucose DMEM medium was removed, neurobasal medium containing 2% B27, 1% glutamic acid, 1% HEPES and 1% penicillin/streptomycin was added and cultured in the 37℃incubator. The neurobasal medium was replaced every three days and cultured for 7–10 days to obtain the primary neurons.

### Cell culture

The primary neurons were cultured in neurobasal medium (Solarbio, China) supplemented with 10% fetal bovine serum (FBS, Hyclone, USA) in a 37℃ incubator.

### Cell transfection

All the plasmids (vector-miR-126a-5pinhibitor and vector-circRNA_0000927over-expression) were purchased from Sangon Biotech. Co., Ltd. (Shanghai, China). The cells were transfected by lipo3000 (Biosharp, China) according to the manufacturer’s protocol. 24-48 h later, cells were used to transfection-efficiency testing via RT-qPCR.

### Hypoxia/reoxygenation cell model

The mouse neuronal injury was simulated by neuron hypoxia/reoxygenation. According to the adjustment of the preliminary experimental results, the time of hypoxia was determined for 3 h and the time of reoxygenation for 24 h, and the process of primary neurons injury in vitro was successfully simulated. The complete DMEM medium was replaced with FBS-free and glucose-free DMEM medium. Then, the cells were placed in an anaerobic producing airbag containing an oxygen indicator, and the bag was filled with mixed gas until the oxygen indicator turned pink. The bag was sealed and placed in an incubator for hypoxia 3 h.After the end of hypoxia, the culture plate was removed from the anaerobic airbag and the medium was updated. The high-glucose DMEM medium containing 10% FBS was replaced and reoxygenated in a normal incubator for 24 h to simulate the injury of primary neurons *in vitro.*

### Flow cytometry

The primary neurons were collected with adjusting the cell density of 1 × 10^6^ cells/mL. Then the cells were washed with PBS two times and centrifugal collected 5 × 10^5^ cells. Next, the cells were resuspended by 500µL binding buffer and 5µLAnnexin V-FITC was added and mixed. Finally, 5µL Propidium Iodide was added and mixed at room temperature and reacted for 15 min in dark. Flow cytometry was used to detect and analyze the proportion of cell apoptosis. The above experiment was repeated for three times and recorded the data.

### Histopathological examination

For Hematoxylin-eosin (HE) staining, the mousebrain tissues were fixed with 4% PFA, dehydrated in gradient ethanol, soaked in xylene, embedded in paraffin, dewaxed wirh xylene and gradient ethanol successively and sectioned. The thickness of the sections was about 3 μm. The slices were sealed with permount TM mounting medium (Genink, Tianjing China,), observed and photographed using a microscope (OlympusBX51, Japan).

### Immunofluorescence (IF)

For immunofluorescence, FITC labeled fluorescent primary antibody NeuN (CST, USA, dilution:1:200) were incubated overnight at 4 °C. On the next day, sections were rewarmed, washed with PBS, anti-rabbit IgG (H + L) Alexa Fluor® 488 Conjugate secondary antibody (CST, USA, dilution:1:500) was incubated in dark for 1 h. After restaining with DAPI, the slices were observed by fluorescence microscope (OLYMPUS, Japan, BX51) and photographed.

### Fluorescence in situ hybridization (FISH)

The mouse brain tissues were fixed with fresh formaldehyde (1–4%) for 1-2 h at room temperature, centrifuged at 12,000 g for 5 min, poured off the supernatant and resuspend the samples with 1xPBS (pH 7.6). The samples were stored in a 1:1 mix of PBS/ethanol at −80 °C until further processing. For the hybridization mixtures, adding 1 volume of probe working solution (50 ng.µL^−1^ DNA) to 9 volume of hybridization buffer in a 0.5-mL microfuge tube and keeping the probe working solution dark and on ice. Hybridization vessels were prepared from 50 mL polyethylene tubes, inserted a piece of blotting paper into a polyethylene tube and soaked it with the remaining hybridization buffer. 10 µL hybridization mix was added to the samples in each well and place the slide into the polyethylene tube (in a horizontal position) and incubated at 46 °C for at least 90 min. The slices were quickly rinsed carefully with a bit of washing buffer, transfered slices into preheated washing buffer and incubated for 25 min at 48 °C. Then the slices were rinsed with distilled H_2_O and counterstained with 10 µL DAPI solution, and incubated for 3 min. Finally, the slices were sealed with permount TM mounting medium (Genink, Tianjing China), observed and photographed using a microscope (OlympusBX51, Japan).

### ELISA assay

The mouse fresh brain tissues and cell samples were added to PBS buffer (Solarbio, China) and ground using a high-throughput tissue homogenizer (Techin TJ-800D, China). The homogenate was centrifuged at 10,000×g for 5 min, and the supernatant was collected. The BCA protein detection kit (Beijing Solarbio Biotechnology Co., Ltd., China) was used for protein quantification. The concentrations of IL-1β (Cloud-Clone, USA) was detected by ELISA kit. The ELISA kit was detected according to the kits instruction.

### Western blotting

The mouse fresh brain tissues and cell samples were ground and lysed in RIPA buffer (Solarbio, China). After tissuesor cell samples lysis, the samples were centrifuged at 12,000 rpm for 10 min at 4 ℃ and supernatants were obtained. The protein concentration was determined using the BCA kit (Solarbio, China). The proteins were denatured by boiling water, and the samples were loaded for SDS-PAGE gel electrophoresis. After electrophoresis, the proteins were transferred to PVDF membrane (Millipore, USA), and the condition was 250 mA constant flow for 1.5 h. Samples were blocked with 5% non-fat milk solution for 1 h and then probed with primary antibodies PERM1 (1:1000, Abcam, USA)、NLRP3 (1:1000, Abcam, USA)、ASC (1:1000, Affinity, USA) and Caspase-1 (1:1000, Abcam, USA) for shaking incubation at 4℃ overnight. After 1×TBST buffer washing for three times, secondary antibody was used for detection included HRP-conjugated anti-rabbit IgG (1:3000, Bioss, China). The samples were incubated for 1 h at room temperature and washed three times with 1×TBST, and incubated with ECL kit (Solarbio, China) for 2 min at room temperature for color development. After ECL kit detection, the automatic chemiluminescence image analysis system (Tanon, China) was used for scanning imaging and taking photos. The software Gelpro32 (Tanon, China) was used to analyze the gray values.

### RT-qPCR

The Trizol reagent (Invitrogen, USA) was used for total RNA of fresh brain tissues and cell samples extraction. The purity and concentration of RNA were detected by NanoDrop and the operation process was performed in strict accordance with the kit operation instructions. The RNA reverse transcription was performed using Revertaid First Strand cDNA Synthesis Kit (Thermo, USA). RT-qPCR was performed using SYBR®Green Kit (TaKaRa, Japan) with GAPDH as the reference gene. The Applied Biosystems Step One Plus Real-Time PCR system (Thermo, USA) was used forCt values detection. Relative expression of target genes was analyzed using Ct values and 2^−△△Ct^values. The primers used in this study were synthesized by BGI (China), and the primer sequences are shown in Table 1.

**Table 1:** Primers used in this study

Gene name		Sequences (5′-3′)	Size(bp)
*lnc_AABR07044470.1*	F	TTAGGCAGGTGGGAGATGATG	81
	R	CTGCAATTATTAAATGCTCATCACTG	
*miR-214-3p*	F	CGCCATTATTACTTTTGGTACGCG	
*PERM1*	F	CTGGGTGGATTGAAGTGGTGTAG	75
	R	TATGTTCGCAGGCTCATTGTTGT	
*GAPDH*	F	GGTGGACCTCATGGCCTACA	82
	R	CTCTCTTGCTCTCAGTATCCTTGCT	
*U6*	F	CTCGCTTCGGCAGCACA	94
	R	AACGCTTCACGAATTTGCGT	

### Double luciferase assay

The binding sites between miR-214-3p and PERM1 mRNA and lnc_AABR07044470.1 and miR-214-3p were predicted basingbioinformatics and Transcriptomic analysis. The deionized water was diluted the 5×PLB before use. Added 50 µL of diluted 1×PLB to each well and shaked on a shaker for 30 min. The 24-well microplate was added with 50 µL of supernatant to each well, and 500 µL of Luciferase Assay Reagent (Boster, China) premixed was added. After the measurement was completed, 450 µL of stop reagent premixed was added to each well and allowed to stand for 5 s, and then the data was measured to determine the intensity of the luciferase reaction. Finally, the results were calculated the ratio of the two sets of data.

### Statistical analysis

The classic scientific software SPSS 24.0 (IBM, USA) was used for data statistical analysis. The 5 replicates of the mice/tissues were used for each set of experiments in the study. The data were expressed as mean ± standard deviation.The *t*-test was used for comparison between the two groups and one-way ANOVA analysis of variance was used among the multiple groups. *P* < 0.05 was considered statistically significant. The analyzed data were plotted using GraphPad Prism 7.0 software (La Jolla, USA).

## Results

### The animal model of AIS is successfully established

**Fig. 1:**
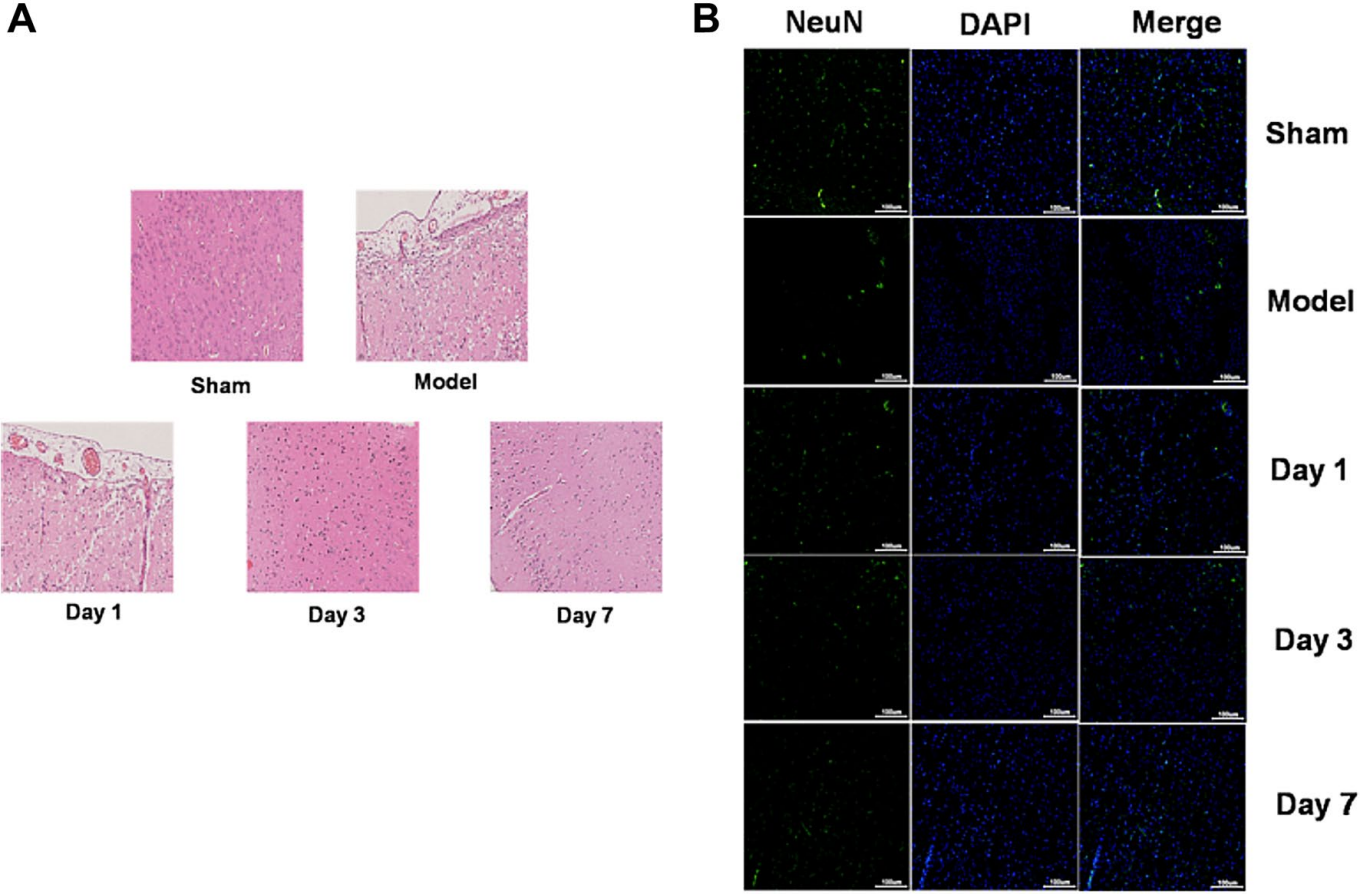
The animal model of AIS is successfully established. **A** HE staining of brain tissues in sham and model groups; **B** IF staining of microglia, astrocyte and neuron in sham and model groups, *n* = 10

In order to confirm the success of the AIS animal model, TTC and HE staining were performed on the brain tissues of mice. TTC staining was used to evaluate the infarction area of mice at1, 3 and 7 days after stroke and the results showed that the infarction area had time-dependent changes with an increase at 1 day after stroke and a gradual decrease at 3 and 7 days after stroke (data not shown). Furthermore, HE staining showed that mouse hippocampus neuron structure was complete and cytoplasmic staining was uniform in the sham group and the pathological changes of rat hippocampal neurons in the model group were obviously, the nuclei were pycnosis, remarkable rupture and interstitial edema at 1 day after stroke and the extent of the lesions was significantly decreased at 3 and 7 days after stroke (Fig. 1A). Moreover, IF analysis using microglial markerIba-1, astrocyte marker GFAP and neuronal marker NeuN, showed that, compared with the sham group, neurons were significantly reduced, while microglia and astrocytes were significantly activated at 1 day after stroke, and this trend gradually recovered at 3 and 7 days after stroke (Fig. 1B), indicating that the animal model of AIS was successfully established and AIS could promote the inflammatory response and neuronal injury.

### PERM1 is the target gene of miR-214-3p

**Fig. 2:**
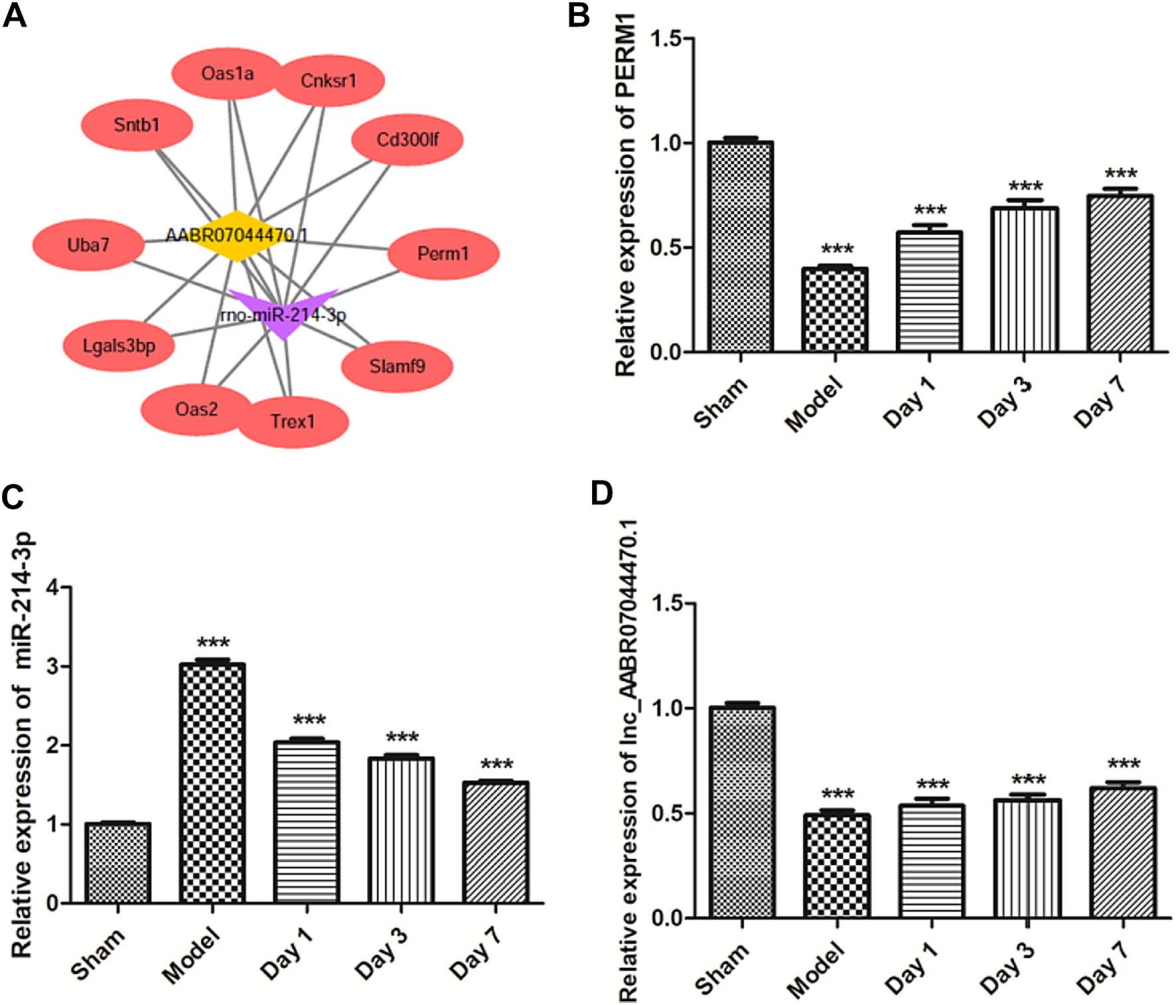
PERM1 is the target gene of miR-214-3p. **A** The ceRNA network bioinformatics prediction; **B** PERM1 expression level was detected in sham and model groups; **C** MiR-214-3p expression level was detected in sham and model groups; **D** lnc_AABR07044470.1expression level was detected in sham and model groups; All data were expressed as mean ± standard deviation, *P < 0.05, **P < 0.01, *** *P* < 0.001 *vs. .*sham group, *n* = 5

In order to further study the molecular mechanism of AIS and neuronal injury, we first analyzed the potential target gene in AIS. The role of PERM1 has been reported in neurodegenerative diseases such as Parkinson’s disease, amyotrophic lateral sclerosis and Alzheimer’s disease [17]. PERM1is involved in the pathophysiology of these diseases and up-regulation or down-regulation of PERM1 expression has a positive or negative impact on the prognosis of the disease [18]. However, the role of PERM1 in the pathophysiology of AIS remains unclear. Therefore, we examined the PERM1 expression in the AIS animal model by RT-qPCR. Interestingly, compared with sham group, PERM1 expression was significantly down-regulated in model group in a time-dependent manner (Fig. 2B). The relationship between PERM1 and miRNA have rarely been reported in cerebrovascular diseases. In order to further study the mechanism of PERM1and miRNA in AIS, firstly, bioinformatics prediction results showed that PERM1 could be combined with miR-214-3p (Fig. 2A). Next, we detected the expression of miR-214-3p in the AIS animal model by RT-qPCR, compared with sham group, miR-214-3p expression was significantly up-regulated in the model group (Fig. 2C).At the same time, we detected the expression of lnc_AABR07044470.1 in the AIS animal model by RT-qPCR, compared with sham group, lnc_AABR07044470.1expression was significantly down-regulated in the model group (Fig. 2D). The results of PERM1 and miR-214-3p showed that they had a negative co-expression correlation and PERM1 was the target miRNA of miR-214-3p.

### MiR-214-3p is the target gene of lnc_AABR07044470.1

**Fig. 3:**
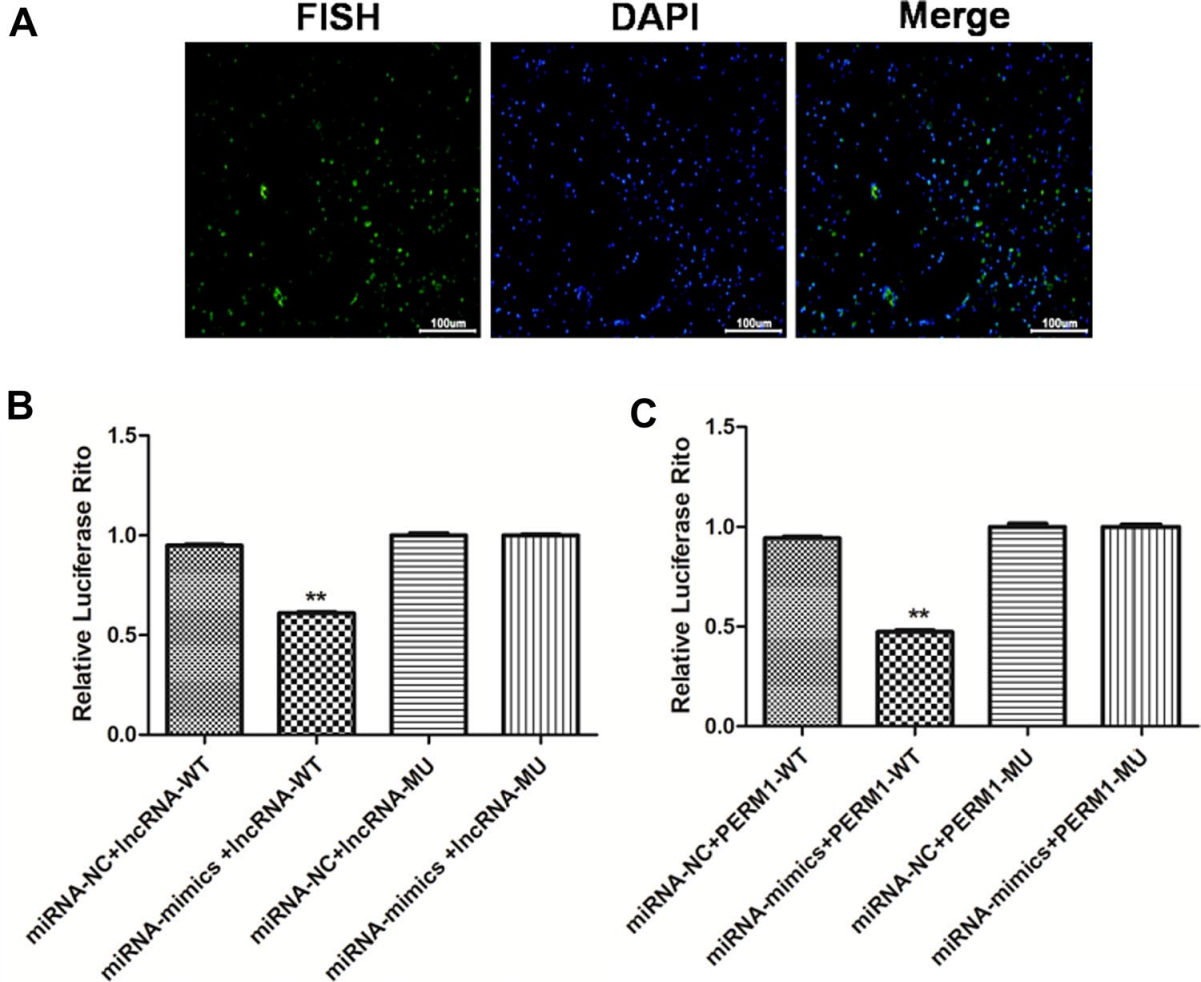
MiR-214-3p is the target gene of lnc_AABR07044470.1. **A** The lnc_AABR07044470.1 localization was detected in sham and model groups. **B** Double luciferase reporter detected lnc_AABR07044470.1 binding to miR-214-3p in model group; **C** Double luciferase reporter detected PERM1binding to miR-214-3p in model group; All data were expressed as mean ± standard deviation, *P < 0.05, **P < 0.01, *** *P* < 0.001vs.sham group, *n* = 5

As is known to all, the research of miR-214-3p focuses on the field of cancer treatment [19], and the research on AIS and neuronal injury is still a blank. In order to further study the molecular mechanism of miR-214-3p in AIS and neuronal injury, we first analyzed the potential target lncRNA binding by miR-214-3p. Expectedly, bioinformatics prediction results showed that lnc_AABR07044470.1 could be combined withmiR-214-3p (Fig. 2A). At the same time, FISH showed that lnc_AABR07044470.1was in the cytoplasm and cytosol in sham and model groups, therefore,lnc_AABR07044470.1 might function as the ceRNA for miR-214-3p (Fig. 3A). Similarly, further detection of double luciferase reporter assay confirmed the binding of lnc_AABR07044470.1 to miR-214-3p (Fig. 3B). Compared with decreased lncRNA-WT group, the relative luciferase activity of lncRNA-MU reporter was not noticeable changed. The results of lnc_AABR07044470.1 and miR-214-3p showed that they had a negative co-expression correlation. At the same time, further detection of double luciferase reporter assay confirmed the binding of PERM1 to miR-214-3p (Fig. 3C). Based on these results, we further speculated that lnc_AABR07044470.1 might relieve the AIS and neuronal injury by inhibiting miR-214-3p expression in vivo. At the same time, we speculated that lnc_AABR07044470.1and PERM1 had a positive co-expression correlation. The results were completely in line with our expectation and lnc_AABR07044470.1would promote the expression of PERM1 (Fig. 3B, C), indicating the lnc_AABR07044470.1 could promote the neuronal injury and AIS via miR-214-3p/PERM1 axis.

### AIS could aggravate the inflammasome formation and activate the inflammatory response in the animal model

**Fig. 4:**
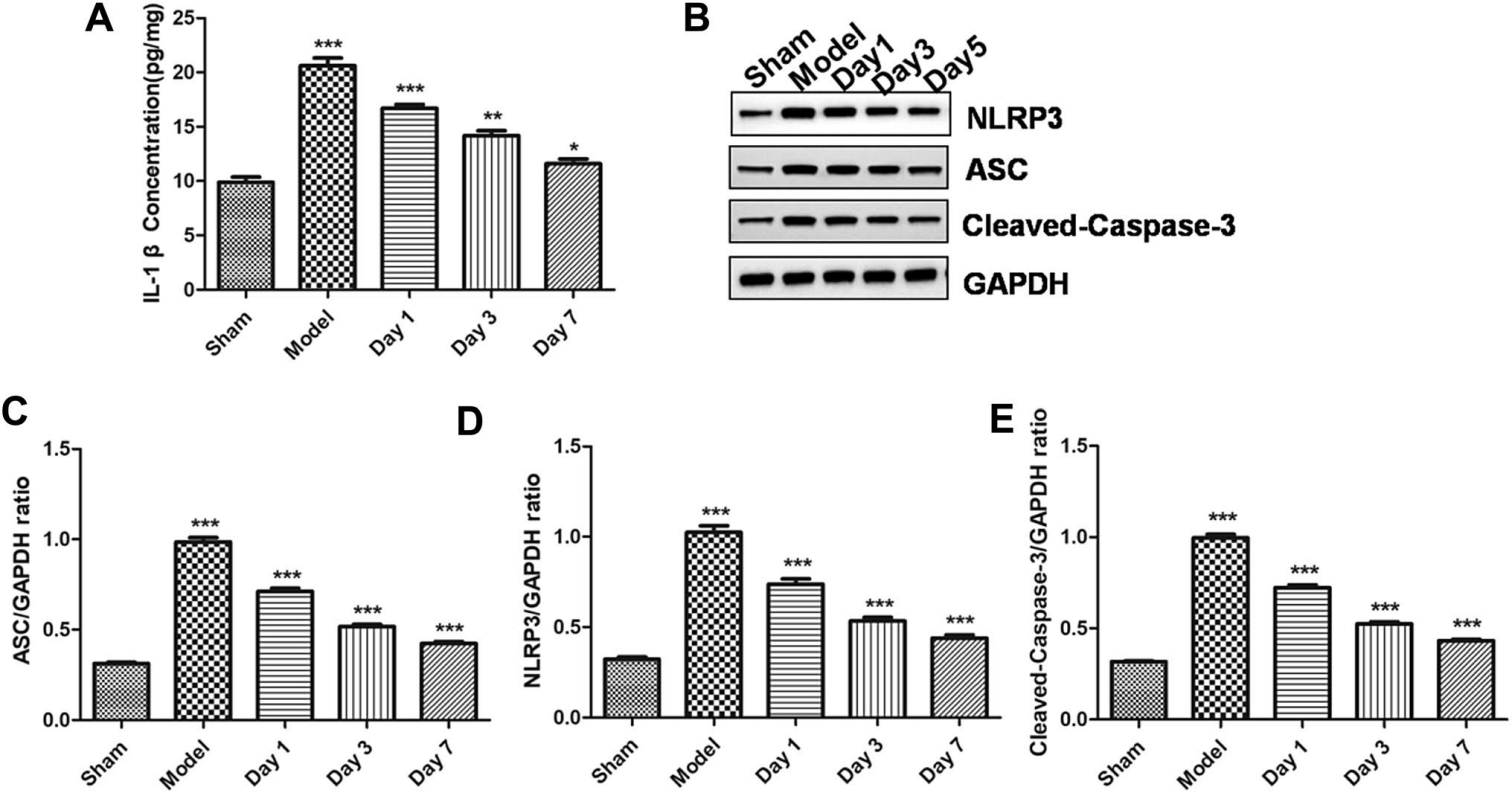
AIS could aggravate the inflammasome formation and activate the inflammatory response in the animal model. **A** Detection of IL-1β levels in different groups; **B**–**E** NLRP3, ASC and Caspase-1 protein expression level was detected indifferent groups. All data were expressed as mean ± standard deviation, *P < 0.05, **P < 0.01, *** *P* < 0.001vs.sham group, *n* = 5

The NLRP3 inflammasome is consists of ASC, Caspase-1 and NLRP3. After receiving the priming signals, NLRP3 inflammasome activationis induced by pathogen-associated molecular patterns (PAMPs) or damage-associated molecular patterns (DAMPs) [2]. Oligomerization of NLRP3 structural proteins binds to PYD of the adaptator protein ASC, and then CARD of ASC binds to CARD on pro-Caspase-1 to form an intact and active NLRP3 inflammasome, which promotes self-cleavage of pro-Caspase-1to produce the active effector protein Caspase-1 [6]. Caspase-1 can cleave the GSDMD to release the N-terminal domain of GSDMD. N-GSDMD can bind to phosphatidylserine and phosphoinositol andpunch on the cell membrane, leading to the imbalance between the inside and outside of the cell and cell death, causing cell contents release and inflammation [7]. In addition to cleaving GSDMD, Caspase-1 can also induce the conversion of IL-1βfrom immature to active state [16]. After cell death, IL-1β will be released out of the cell to induce the neuro-inflammation [20]. Therefore, inflammatory response and NLRP3 inflammasome can be considered as markers of AIS. Firstly, compared with sham group, the level of IL-1β was also significantly increased in the model group (Fig. 4A). Secondly, compared with sham group, the protein expression of NLRP3, ASC and Caspase-1 was significantly increased in the model group (Fig. 4B–E), indicating that AIS could aggravate the inflammasome formation and activate the inflammatory response in the animal model. Therefore, based on these results, we further speculated that lnc_AABR07044470.1could promote the inflammatory response to neuronal injury viamiR-214-3p/PERM1 axis in the AIS animal model.

### Over-expression of lnc_AABR07044470.1 and inhibition of miR-214-3p relieve the neuronal injury in the hypoxia/reoxygenation cell model

**Fig. 5:**
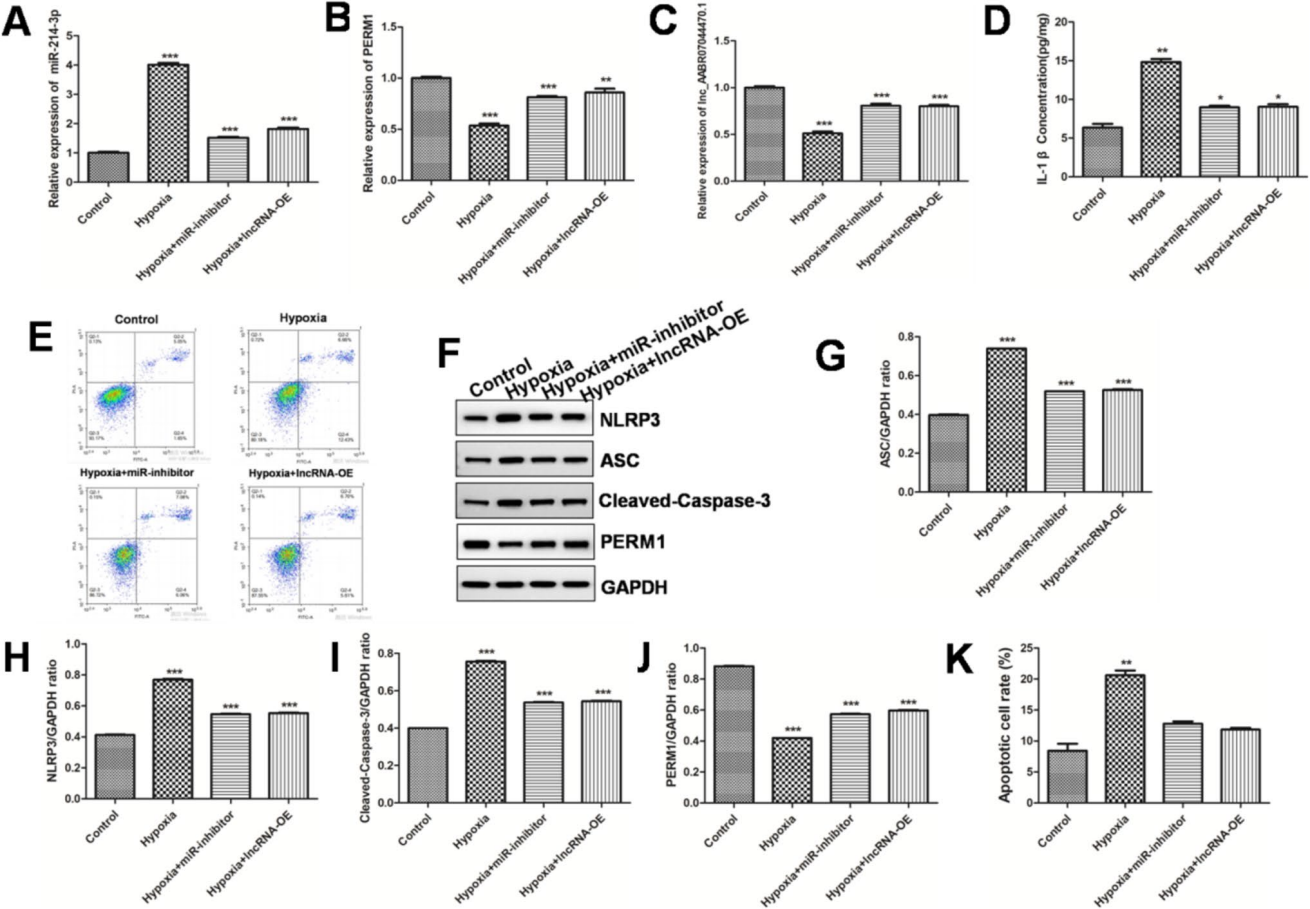
Over-expression of lnc_AABR07044470.1 and inhibition of miR-214-3p relieve the neuronal injury in the hypoxia/reoxygenation cell model (A–C) Detection of vector-miR-214-3p inhibitor and lnc_AABR07044470.1 over-expression vectors transfection efficiency in the hypoxia/reoxygenation cell model; (D) Detection of IL-1β levels inthe hypoxia/reoxygenation cell model; (E, K) Neuron apoptosis was detected by flow cytometry in the hypoxia/reoxygenation cell model; (F–J) NLRP3, ASC, Caspase-1 and PERM1 expression level was detected in the hypoxia/reoxygenation cell model. All data were expressed as mean ± standard deviation, *P < 0.05, **P < 0.01, *** *P* < 0.001vs.control group, *n* = 5

Whether the experimental results in vitro are consistent with the AIS animal model is a question we are very concerned about, in order to find out the mystery, we successfully established the primary neuron model with hypoxia/reoxygenation treatment and transfected vector-miR-214-3p inhibitor and vector-lnc_AABR07044470.1 into the hypoxia/reoxygenation cell model. Compared with control and model groups, the RT-qPCR results indicated that vector-miR-214-3p inhibitor transfection significantly down-regulated the expression of miR-214-3pand up-regulated the expression of PERM1 and vector-lnc_AABR07044470.1 over-expression transfection significantly up-regulated the expression of lnc_AABR07044470.1 and PERM1 in the primary neuron (Fig. 5A–C). Furthermore, we detected the expression of IL-1β by ELISA assay. Compared with model group, the level of IL-1βin the vector-miR-126a-5p inhibitor and lnc_AABR07044470.1 over-expression groups was significantly decreased (Fig. 5D). Moreover, cell apoptosis was detected using TUNEL assay. Interestingly, compared with model group, less apoptosis cells were observed in vector-miR-214-3p inhibitor and lnc_AABR07044470.1 over-expression groups (Fig. 5E, K). At the same time, compared with model group, the expression of NLRP3, ASC, Caspase-1 was significantly down-regulated and PERM1 was significantly up-regulated in the vector-miR-214-3p inhibitor and lnc_AABR07044470.1 over-expression groups (Fig. 5F–J), this was consistent with the experimental results of the AIS animal model, indicating that over-expression of lnc_AABR07044470.1 and inhibition of miR-214-3p expression could relieve the neuronal injury in the hypoxia/reoxygenation cell model.

## Discussion

Stroke refers to the lesion that blood perfusion or oxygen supply is restored to the brain tissue after a period of ischemia, and the damage caused by ischemia to the tissue is not alleviated, and even continues to worsen [21]. Stroke is the main pathophysiological process of brain dysfunction in most ischemic cerebrovascular diseases. Stroke can be divided into three stages [22]: (1) Acute phase: occurs within a few minutes or hours after cerebral ischemia, which is caused by the necrosis of nerve cells and brain tissue injury directly; (2) Subacute phase: occurs several days after brain ischemia, that is, the molecular cell damage stage. Excitatory amino acid toxicity, cell apoptosis, calcium overload, NO toxicity, oxygen radicals, mitochondrial dysfunction and energy metabolism disorders are involved in the brain tissue injury at this stage. (3) Delayed injury period: occurs several days to several weeks after cerebral ischemia, vasogenic edema and a large number of inflammatory reactions occur in the brain tissue, which further aggravate the damage of nerve cells [23]. Among them, AIS is the most lethal pathological stage. Therefore, it is of great significance to find effective ways to understand the molecular mechanism for the effective AIS treatment and the neuroprotection of the ischemic brain after stroke.

Until now, lncRNA has been recognized as an important type of non-coding RNA, an important regulator of cellular processes, and an active participant in various signal transduction pathways [24]. There is increasing evidence that they regulate various cellular functions, such as cell proliferation, cell growth, cell migration, stem cell maintenance, epithelial mesenchymal transformation, and apoptosis [25]. In addition, the study of the interactions between lncRNA and other biomolecules, such as lipids and second messengers, will further elucidate the biological functions of lncRNA under physiological or pathological conditions.The potential clinical application prospect of lncRNA is huge [9]. LncRNA is often abnormally expressed and expressed in cerebrovascular disease and high tissue specificity [26]. Therefore, targeted therapy targeting lncRNA may be developed into a new way to treat cerebrovascular disease in the future, and theoretically, it may not cause the harmful side effects to healthy tissues [27]. In the traditional clinical diagnosis and treatment, imaging diagnosis, such as CT, MRI and other technologies as well as the application of disease markers, for the diagnosis and treatment of AIS patients provide a certain guiding role [28]. However, traditional examination still have great limitations in accurately predicting the occurrence and development of diseases. LncRNA is widespread and relatively stable in organisms, and can be detected in body fluids such as blood and urine, so it has the possibility of being used as a clinical marker [3].In addition, the novel studies showed that lncRNAs can bind specific target genes and regulate their functions, thus affecting the occurrence and progression of diseases.

In this study, based on bioinformatics prediction, we screened out several lncRNAs that were differentially expressed in AIS, among which the most significant lncRNAs waslnc_AABR07044470.1.To study the function of lnc_AABR07044470.1. Next, we used bioinformatics database to construct the interaction network between lncRNA and miRNA. At this point, we basically determined that lnc_AABR07044470.1may play an important biological role in the occurrence and development of AIS. To verify our prediction, we performed the RT-qPCR in AIS animal model, and the results showed that lnc_AABR07044470.1 was significantly down-regulated in the AIS animal model, so it is reasonable to believe that our study is of great significance. So far, many literatures have reported that lnc_AABR07044470.1 plays an important role in a variety of tumors [29], but the role of lnc_AABR07044470.1 in AIS is still unsolved. Our study found that the expression of lnc_AABR07044470.1 was significantly decreased in the AIS animal model, and circRNA_0000927 could promote the development and deterioration of AIS by binding to target miRNA. The mechanism of lnc_AABR07044470.1 is not yet fully understood, but our study suggests that lnc_AABR07044470.1 has the potential to inhibit and mitigate the occurrence and development of AIS and may be a novel therapeutic target for AIS.

MiR-214-3p, as a novel member of the miRNA family, has been found to be involved in traumatic fractures of the brain, tumors, osteoarthritis, endothelial cell injury of coronary heart disease, myocardial cell fibrosis and other aspects [30]. However, the role of miR-214-3p in AIS and neuronal injury has not been reported. In this study, we confirmed that miR-214-3p was up-regulated in the process of AIS in animal model, and bioinformatics also predicted that miR-214-3p, as a target gene of lnc_AABR07044470.1, was involved in the regulation of the development of AIS. After transfection with miR-214-3p inhibitor and over-expresson lnc_AABR07044470.1 vectors, it was found that the expression of NLRP3, ASC, Caspase-1 and PERM1 was decreased after miR-214-3p inhibition and over-expresson lnc_AABR07044470.1. At the same time, after miR-214-3p was inhibited, the survival rate of primary neurons was increased and the apoptosis rate of primary neurons was decreased. Therefore, our experiment confirmed that miR-214-3p plays a crucial role in the process of AIS, and the promotion of miR-214-3p on AIS may be realized by regulating the effect of primary neurons apoptosis.

Previous studies found that PERM1 had protective effects in primary neurons and human astrocytes cultured in vitro [5, 31]. Over-expression of PERM1 in neurons could inhibit the neuronal apoptosis, while inhibition of PERM1 expression could induce the neuronal apoptosis [6]. It was found that the expression of PERM1 and related antioxidant components were increased in AIS patients, and the increased level of PERM1 was mainly located in activated astrocytes [2, 19]. By over-expressing PERM1 in astrocytes in vitro, ROS levels were reduced, pro-inflammatory factor release was reduced, and neuronal activity was ultimately improved. These results suggested that PERM1 in astrocytes might act as an endogenous protective mechanism in AIS patients [32]. PERM1 is also involved in regulating the mitochondrial metabolism system of astrocytes, which in turn reduces the oxidative and metabolic damage [33]. Considering these neuro-protective effects of PERM1, regulating the level of PERM1 could be a promising neuro-protective strategy [34].In this study, we reported the previously unknown specific role of lnc_AABR07044470.1-miR-214-3p-PERM1 axis in the pathophysiology of AIS, which could provide a new theoretical basis for revealing the molecular mechanism of neuro-protection of PERM1.

## Conclusion

In summary, vital lncRNAlnc_AABR07044470.1 is down-regulated in AIS animal and cell models and is related to prediction and treatment of the occurrence and development of AIS. Lnc_AABR07044470.1 promotes the AIS occurrence and deterioration and neuronal injury by targeting the miR-214-3p and PERM1 and activating the mitochondrial-damaged inflammatory response.

## Data Availability

The data and materials involved in this manuscript were original results. If the magazine or readers need the data and materials, we can also upload all the data and materials to provide online. The authors declared that all relevant data and materials were available within the paper on reasonable request.
